# Chromatin immunoprecipitation (ChIP) method for non-model fruit flies (Diptera: Tephritidae) and evidence of histone modifications

**DOI:** 10.1371/journal.pone.0194420

**Published:** 2018-03-15

**Authors:** Kumaran Nagalingam, Michał T. Lorenc, Sahana Manoli, Stephen L. Cameron, Anthony R. Clarke, Kevin J. Dudley

**Affiliations:** 1 School of Earth, Environmental and Biological Sciences, Queensland University of Technology (QUT), Brisbane, Qld, Australia; 2 Department of Entomology, Purdue University, West Lafayette, IN, United States of America; Ludwig-Maximilians-Universitat Munchen Adolf-Butenandt-Institut, GERMANY

## Abstract

Interactions between DNA and proteins located in the cell nucleus play an important role in controlling physiological processes by specifying, augmenting and regulating context-specific transcription events. Chromatin immunoprecipitation (ChIP) is a widely used methodology to study DNA-protein interactions and has been successfully used in various cell types for over three decades. More recently, by combining ChIP with genomic screening technologies and Next Generation Sequencing (e.g. ChIP-seq), it has become possible to profile DNA-protein interactions (including covalent histone modifications) across entire genomes. However, the applicability of ChIP-chip and ChIP-seq has rarely been extended to non-model species because of a number of technical challenges. Here we report a method that can be used to identify genome wide covalent histone modifications in a group of non-model fruit fly species (Diptera: Tephritidae). The method was developed by testing and refining protocols that have been used in model organisms, including *Drosophila melanogaster*. We demonstrate that this method is suitable for a group of economically important pest fruit fly species, viz., *Bactrocera dorsalis*, *Ceratitis capitata*, *Zeugodacus cucurbitae* and *Bactrocera tryoni*. We also report an example ChIP-seq dataset for *B*. *tryoni*, providing evidence for histone modifications in the genome of a tephritid fruit fly for the first time. Since tephritids are major agricultural pests globally, this methodology will be a valuable resource to study taxa-specific evolutionary questions and to assist with pest management. It also provides a basis for researchers working with other non-model species to undertake genome wide DNA-protein interaction studies.

## Introduction

Interactions between DNA and associated proteins drive the transcriptional regulation of genes [1]. For example, post-translational modifications of histone proteins play a key role in regulating functional genes that are represented as phenotypic characteristics in multicellular organisms [2, 3]. Covalent modifications of DNA-bound histone proteins are thought to be important mediators of this process by directly influencing DNA conformation, as well as acting as signals for other DNA-interacting proteins to bind. Histones are the chief components of chromatin, which directly influence gene regulation by altering the packaging of DNA [4]. In addition to gene regulation, histones and associated chromatin proteins are involved in the repair, recombination and replication of DNA [3]. The post-translational histone modification states (as well as epigenetic DNA modification states in many organisms) that are found across the genome of particular cell population is collectively known as the epigenome. Profiling of the epigenomes is crucial to understanding the cellular and biological processes controlled by functional genes [5].

The technique predominantly used to profile the chromatin component of the epigenome is chromatin immunoprecipitation, followed by sequencing (ChIP-seq) [6, 7]. ChIP-seq has been effectively used to characterize DNA sequences associated with specific transcription factors and post-translational histone modifications in many model systems [8–12]. Briefly, ChIP-seq involves *in vivo* cross-linking of proteins that are bound to DNA, followed by lysing cross-linked chromatin from cells, and fragmentation of chromatin material to a desirable sized product (200–1000 bp) for downstream analyses [13]. Cross-linked chromatin fragments are then immunoprecipitated by conjugation with antibodies that recognize specific protein or protein modifications present in the chromatin [5]. Finally, DNA is released from the immunoprecipitated chromatin by reverse cross-linking, and this DNA is then sequenced to determine the genomic regions that were originally bound by the protein or protein modification of interest [14].

While genome wide profiling of ChIP DNA has been central to the study of DNA-protein interactions for over a decade, application of ChIP has been inherently limited to the well characterised model species. This is largely because ChIP profiling methods are complex, meaning that methodologies developed for model systems cannot easily be employed directly in non-tested systems [6]. One reason for this complexity is that anatomical characteristics of tissue material can have a large impact on sample processing efficiency. Indeed, when we tested ChIP methods published on the antibody supplier Abcam’s website (which recommends using liver tissue as starting material), as well as a published method designed for *Drosophila* testis cells [15], the result was inefficient recovery of ChIP DNA from the sclerotized head tissue of tephritid fruit flies. There are numerous other ChIP publications available [14–18]; however, testing every component of all these methods is an expensive and time-consuming task. It is therefore desirable to create ChIP-seq methods that have been shown to work in non-model systems.

We report here a method that can be successfully applied for genome wide profiling of post-translational histone modifications (e.g. ChIP-seq) in non-model tephritid fruit flies. This method has been devised by amalgamating and revising a number of previously published methods [15, 17], as well as testing for the first time in tephritid fruit flies five commercially available antibodies that target various well known covalent histone modifications (i.e. histone 3 lysine 4 trimethylation (H3K4me3), histone 3 lysine 27 trimethylation (H3K27me3), histone 3 lysine 27 acetylation (H3K27ac), histone 3 lysine 36 monomethylation (H3K36me1), and histone 3 lysine 36 trimethylation (H3K36me3)). These histone modifications are connected to various gene functions: H3K4me3 modification occurs at transcription start site of active genes, while H3K27 acts in opposition to H3K4me3 and is associated with shutting down transcription [19]. Modification in H3K36 explains many molecular functions including repression of transcription, alternative splicing and DNA repair, and biological processes such as longevity [20, 21]. We successfully implemented this method in a number of major pest species, namely the Oriental fruit fly, *Bactrocera dorsalis* (Hendel), the Mediterranean fruit fly, *Ceratitis capitata* (Weidemann), melon fly, *Zeugodacus cucurbitae* (Coquillet) and the Queensland fruit fly, *Bactrocera tryoni* (Froggatt). In addition to the methodology, we also report here, for the first time, evidence of histone modifications across the genome of *Bactrocera* fruit flies. While histone modification through immunodetection assay has been attempted in *Anastrepha fraterculus* [22], our study has evidenced genome wide modifications in a tephritid species identified through ChIP-seq.

Tephritid fruit flies are globally important pests of fruit and vegetable crops, and are highly invasive with complex reproductive behaviours [23–26]. Additional to their pest status, tephritids are also used as models in evolutionary biology, e.g. the apple maggot fly, *Rhagoletis pomonella* (Walsh), is the test book organism for sympatric speciation [27]. While high throughput genetic approaches have advanced our understanding of developmental, behavioural and physiological processes in these flies [28–34], modern tools such as ChIP-seq have not previously been employed in tephritid fruit flies. As in other organisms, epigenomic profiling using techniques such as ChIP-seq is critically important to understand transcriptional regulation of important biological and physiological processes, such as mating and development, for better pest management and to understand ecological processes in tephritids [24, 35]. Therefore, the method reported here will be an important resource for researchers focusing on this important group of non-model organisms. In addition, it is likely that this method will be useful for other researchers with an interest in studying DNA-protein interactions in tissue material of a similar composition (e.g. in sclerotized head tissue) to that tested here.

## Materials and methods

### Insect source

*Bactrocera tryoni* was sourced from a colony maintained at the [Queensland] Department of Agriculture and Forestry, Brisbane, Australia. The colony was refreshed annually by introducing wild flies from cultivated fruits. Live flies were killed by chilling and used for the downstream analysis. *Ceratitis capitata* was obtained from a colony maintained at the [Western Australian] Department of Primary Industries and Regional Development, Perth, Australia. *Bactrocera dorsalis* and *Z*. *cucurbitae* were collected from infested fruits in Thailand and reared at the Plant Protection Research and Development office, Department of Agriculture, Thailand. *Ceratitis capitata*, *B*. *dorsalis* and *Z*. *cucurbitae* were snap frozen as sexually matured adults and stored in RNAlater at -80°C until further analysis.

### Procedure

#### Crosslinking and cell lysis

For crosslinking, we used a total of 30-40mg tissue (heads of 30 flies per tube) and dissected out in cold phosphate-buffered saline (PBS) with 1x protease inhibitor (30 μL /ml). Larger material as suggested in generic protocols (1 g) can result in poor crosslinking. The tissue was then rinsed twice with PBS and re-suspended in 200 μL of the same PBS solution with protease inhibitors. To crosslink the protein with DNA, 5.5 μL of 37% formaldehyde was added to tissue and incubated at room temperature (RT) for 5 minutes by vortexing in between. Crosslinking using formaldehyde is reversible, however over crosslinking can affect the fragmentation and ultimately the efficiency of immunoprecipitation and needs to be stopped. Hence, 0.125M glycine was added, incubated at RT for 5 minutes and vortexed in between to stop the crosslinking. PBS from tissue was removed and discarded, and the tissue was rinsed twice in 450 μL of PBS with protease inhibitor (The cross-linked tissues can be stored at—20°C at this point).

To lyse the cells and get unicellular suspension, tissue was suspended in 200 μL of lysis buffer (please refer Table 1 for buffer recipes) with protease inhibitor added fresh to the buffer each time, and homogenized using a bench-top mixer mill (Retsch® mixer mill MM 400). The lysed tissues were incubated at RT for 10 minutes.

**Table 1:** Buffers used for cell lysis and immunoprecipitation for ChIP sequencing of tephritid fruit flies.

Buffer	Recipe
Lysis buffer	50mM Tris-HCl pH 8.0, 1mM CaCl_2,_ 0.2% Triton X-100, 5mM Sodium Butyrate, Protease inhibitor cocktail
RIPA buffer	10mM Tris-HCl pH 8.0, 1mM EDTA pH 8.0, 0.1% SDS, 0.1% Sodium Deoxycholate, 1% Triton X-100, Protease inhibitor cocktail
IP Dilution buffer	1% Triton X-100, 20mM Tris-HCl pH 8.0, 2mM EDTA pH 8.0, 150mM NaCl (add last), Protease inhibitor cocktail
Wash buffer	0.1% SDS, 1% Triton X-100, 20mM Tris-HCl pH 8.0, 2mM EDTA pH 8.0, 150mM NaCl (add last)
Final wash buffer	0.1% SDS, 1% Triton X-100, 20mM Tris-HCl pH 8.0, 2mM EDTA pH 8.0, 500mM NaCl (add last)
Elution buffer	1% SDS, 100mM NaHCO_3_

#### Shearing

For an effective ChIP, chromatin DNA of 200–300 bp is required which allows profiling of histone modifications at a resolution of approximately one to two nucleosomes. The sonication procedure described below sheared the chromatin to the size of 200–300 bp for tephritid fruit flies. For a larger size fragments, and for softer tissues sonication duration may be reduced. To fragment the chromatin DNA, the lysate was transferred into sonication tubes (0.5 ml) and sheared using the following setting: 1.25 to 1.5 h at high settings; 30 sec on: 30 sec off cycle (This setting is for Bioruptor® UCD-200 and the condition may vary for other types of sonicators. During sonication, the water tank was topped-up with ice as shearing by keeping the DNA product in ice-cold water rather than ice can yield poor shearing. After sonication, the cell lysate was centrifuged at 4°C for 30 sec at 8000 rcf to remove the insoluble cellular debris (It was ensured that the centrifuge is ready at 4°C for spinning prior to this step). The supernatant containing sheared chromatin DNA was transferred to a new tube and diluted by adding 1 ml of RIPA buffer (protease inhibitor added to RIPA buffer fresh each time).

#### Input DNA

For quality checks, DNA quantification and downstream analysis, 40 μL of diluted chromatin was aliquoted and used as input. Then 2 μL of 5M NaCl was added to the aliquot and incubated at 65°C overnight (O/N). After the O/N incubation, 2 μL RNAse A was added to the aliquot and incubated at 65°C for 2 h. Then 5 μL of proteinase K was added and incubated at 65°C for 4 h. The chromatin DNA was purified using Bioline® DNeasy minikit following manufacturer’s protocol. Input samples were not conjugated with any antibodies and hence served as ‘control’ to compare any histone modifications in antibody conjugated samples.

#### Bead preparation

Protein A/G beads at the rate of 20 μL per immunoprecipitation are recommended. Protein beads were washed three times in 3X immunoprecipitation dilution buffer (IP dilution buffer). Tubes were applied to magnet and the dilution buffer was aspirated off and discarded. To the beads, 75 ng (per μl beads) of Herring Sperm DNA, 0.1 μg (per μl beads) of Bovine Serum Albumin and twice the bead volume of dilution buffer was added followed by incubation for 30 minutes with rotation at RT. The beads were washed once with 3X IP dilution buffer, and resuspended in twice the bead volume of IP buffer.

#### Pre-clearing of chromatin and antibody conjugation

Pre-clearing of chromatin with magnetic beads helps to remove the non-specific proteins that can negatively affect immunoprecipitation. For pre-clearing, 20 μl of beads was added to each chromatin sample and incubated at 4°C for 1 hr with rotation. To the 20 μl beads, 5–10 μl of antibody of interest (please refer Table 2 for antibodies information) and 75 μl PBS was added, and incubated at 4°C for 4 hrs (incubation can be performed at RT for 1 hr, however, for effective antibody conjugation to beads, incubating at 4°C is recommended). For mock IP (control), only PBS was added.

**Table 2:** List of antibodies used for protocol optimization and their product codes for chromatin immunoprecipitation sequencing of tephritid fruit flies.

	Product code (Abcam)	Antibody (ChIP grade)
1	ab9050	Rabbit polyclonal to Histone H3 (tri methyl K36)
2	ab9048	Rabbit polyclonal to Histone H3 (mono methyl K36)
3	ab8580	Rabbit polyclonal to Histone H3 (tri methyl K4)
4	ab7766	Rabbit polyclonal to Histone H3 (di methyl K36)
5	ab6002	Mouse monoclonal to Histone H3 (tri methyl K27)
6	ab4729	Rabbit polyclonal to Histone H3 (acetyl K27)
7	ab1791	Rabbit polyclonal to Histone H3 (Pan-H3)

#### Immunoprecipitation

The beads with antibody were applied to magnet and the supernatant was discarded. The beads with sample was applied to magnet and the supernatant was aspirated off and added to the antibody conjugated beads, which was followed by incubation at 4°C O/N with rotation. The immunoprecipitated chromatin sample was applied to magnet and the supernatant was discarded. Then the immunoprecipitate was washed quickly with 1 ml of wash buffer (low-salt buffer) for 1 min twice followed by another wash for 5 minutes once with wash buffer. The precipitate was again washed with final wash buffer (high-salt) for 1 minute followed by two minutes more. So totally three washes (1-1-5 minutes) with low-salt wash buffer and two washes (1–2 minutes) with high-salt buffer was performed. After the final wash, beads were applied to magnet and buffer was aspirated off and discarded.

#### Elution and reverse crosslinking

To elute the DNA, 120 μl of elution buffer was added to beads and rotated for 15 minutes at RT. The beads were applied to the magnet and the supernatant was transferred to new tube. To the eluted DNA, 2 μl (per 40 μl sample) of 5M NaCl was added and incubated at 65°C O/N. Then 2 μl per 40 μl sample of RNAse A was added and incubated for 2 h at 65°C O/N. To RNAse treated DNA, 5 μl per 40 μl sample of proteinase K was added and incubated for 4 h at 65°C O/N. Then the DNA was purified using Bioline® DNeasy minikit following manufacturer’s protocol and used for sequencing.

#### Library preparation and sequencing

Library preparation was performed using Illumina TruSeq ChIP sample preparation kit following manufacturer’s protocol. Sequencing was performed using the Illumina Nextseq 500 platform located at the Central Analytical Research Facility, Queensland University of Technology, Brisbane, Australia. Paired end sequencing (2 x 40 bp) was used to generate sequence data, with at least 20 million reads sequenced per library.

The reagents and equipment used in various steps of chromatin immunoprecipitation and sequencing are listed in S1 Table.

## Results and discussion

We modified available ChIP methodologies in steps crosslinking, shearing and during antibody conjugation and the amount of starting material used, and this was shown to work for *B*. *tryoni*. These modifications subsequently worked for other tephritids *viz*., *B*. *dorsalis*, *C*. *capitata* and *Z*. *cucurbitae* without further modification. Using the modified methodology, we generated quality chromatin DNA for sequencing. We checked the cross-linked chromatin extractions prior to and after conjugating antibodies using Bioanalyzer 2100 and agarose gel electrophoresis, and the results showed that the method effectively bound DNA-associated proteins with DNA *in situ*.

**Fig 1:**
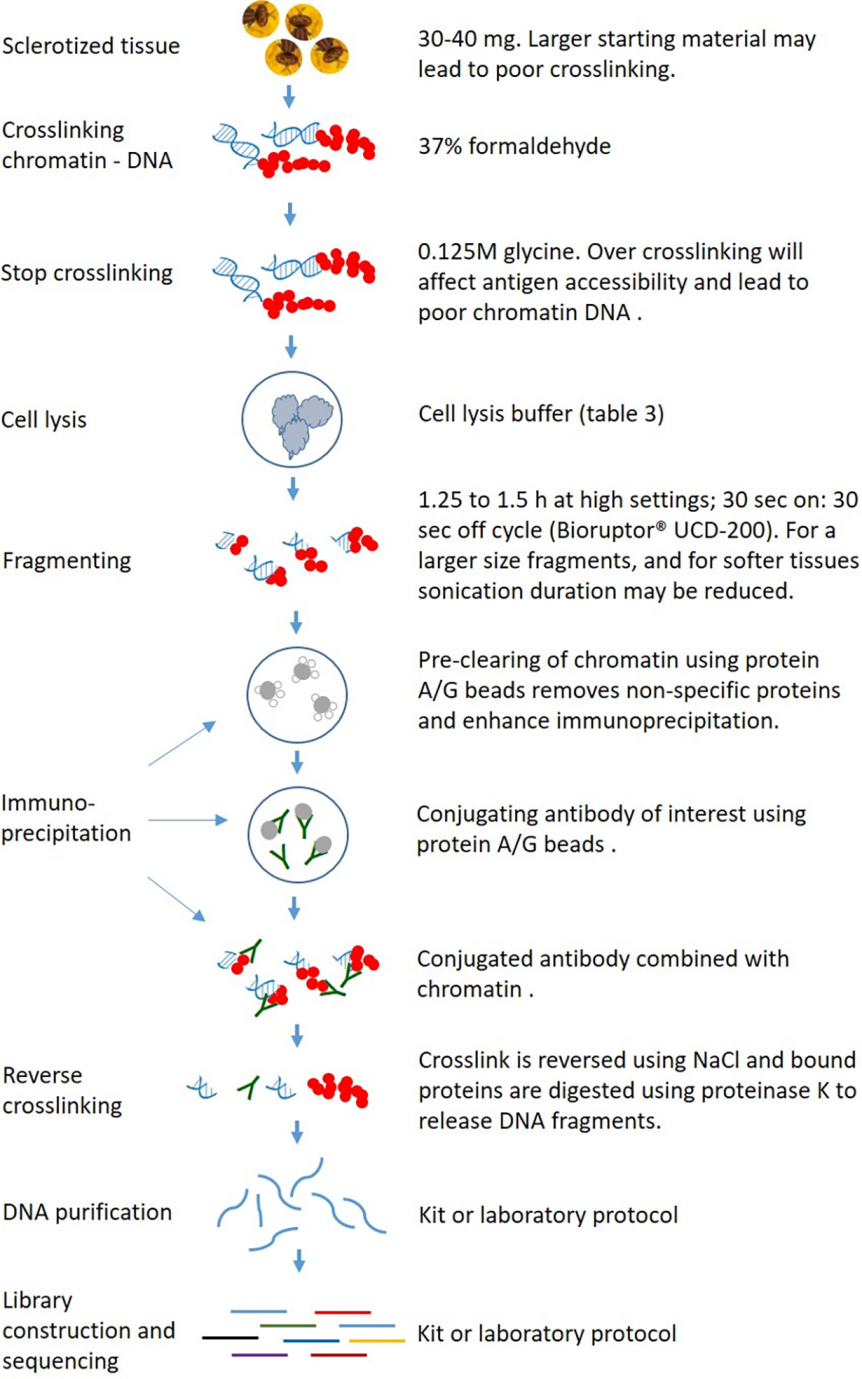
Schematic representation of ChIP workflow and crucial steps for an effective crosslinking and immunoprecipitation.

With the *Drosophila* method [15], we could not generate quality chromatin DNA, perhaps because of over cross-linking. Cross linking is crucial for successful ChIP, and over cross-linking will affect antigen accessibility and lead to poor chromatin DNA which ultimately affects immunoprecipitation [14]. Hence, we integrated a published procedure of adding 0.125M glycine (Fig 1)into our methodology which stopped over cross-linking [17]. Further, the more general Abcam method resulted in poor cross-linking perhaps because of larger starting material (1 g tissue). We used only 30–40 mg of head tissues as starting material per tube, which yielded sufficient chromatin DNA for at least two immunoprecipitations. A simplified summary of the key steps in our ChIP workflow, noting particularly components where we initially encountered problems which required optimisation, is provided in Fig 1.

**Fig 2:**
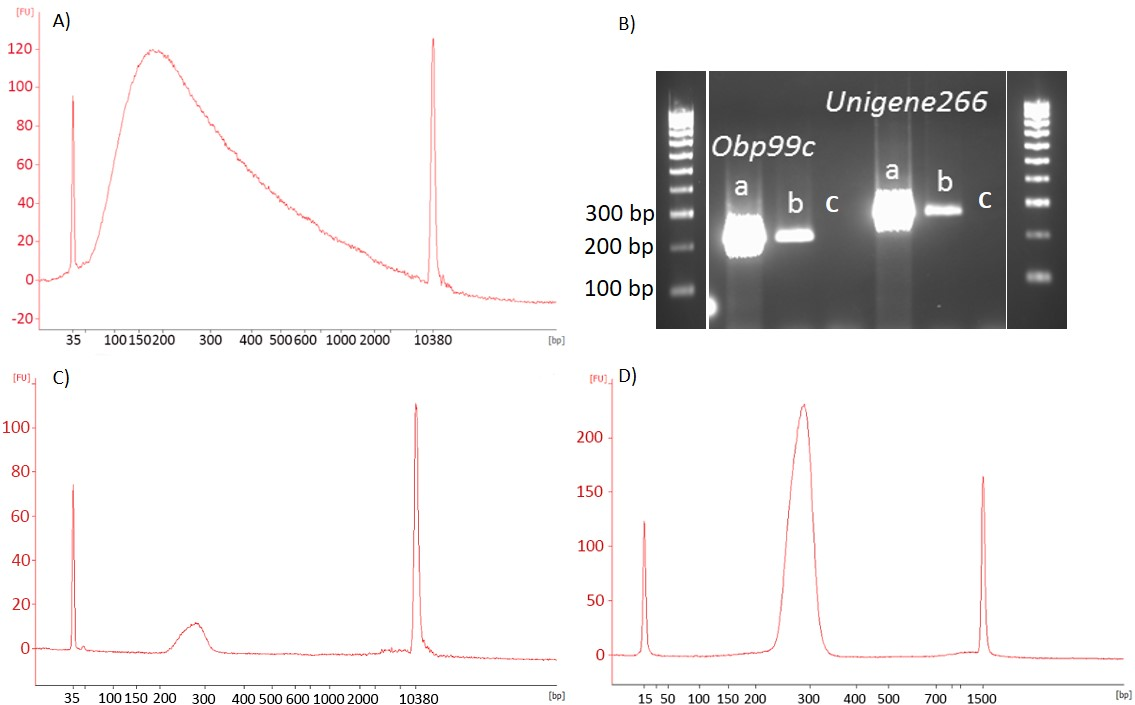
(A) An example of product size after sonication using Bioruptor at 30 sec on, 30 sec off at high speed for 70–80 minutes; (B) Conjugation of (a) H3K27me3 (b) H3K27 acetylation (c) mock in *B*. *tryoni* checked for *Obp 99c* and *Unigene266* genes; (C) Bioanalyzer image showing the size and concentration of chromatin DNA immunoprecipitated with H3K27me3 (D) Chromatin DNA immunoprecipitated with H3K27me3 after enrichment.

Following generation of chromatin DNA, the next crucial step in ChIP is shearing. Chromatin DNA needs to be fragmented into short fragments and released from the cell nuclei to allow antibodies to conjugate effectively. A fragment size of 200–300 bp also allows profiling of histone modifications at a resolution of approximately one to two nucleosomes (as 147 nucleotides of DNA wrap around a single nucleosomal unit). For these reasons, it is important to check the DNA size prior to immunoprecipitation. Shearing is an onerous step when performing the assay for first time since product sizes need to be checked at regular intervals to optimize the duration. While shearing is exclusively dependent on the type of instrument used, poor shearing of fruit fly tissues was noticed when we followed available protocols. The Bioruptor setting described above in the methodology (Fig 1) fragmented tephritid chromatin to a required size of 200–300 bp (Fig 2A). In this assay, we used the heads of the flies which are a heavily sclerotized body part; softer tissues may perhaps need shorter duration of sonication. For most fruit fly tissues, the 1.25 to 1.5 h timing window could be the upper limit for fruit flies.

Conjugating antibody with chromatin DNA is the next critical step for an effective ChIP, and numerous factors (e.g. cross-linking, larger DNA fragments and poor quality antibodies) can contribute to poor immunoprecipitation. We incorporated a step (Fig 1) prior to antibody conjugation to do pre-clearing of chromatin sample which helped to remove the non-specific proteins. We checked our immunoprecipitation using Qubit and bioanayzer profiles. The data showed high quality DNA of 200–300 bp size (Table 3), and an effective antibody conjugation which was further confirmed using *Obp99* and *unigene266* primers in *B*. *tryoni* (Fig 2B, 2C & 2D). The Abcam method required 25 μg of chromatin DNA per immunoprecipitation for an effective conjugation; however we used only 240–340 ng which yielded immunoprecipitated DNA of 50–195 ng depending on antibodies used (Table 4), which is within the range recognized as optimum for a ChIP assay [6]. Using 10 ng immunoprecipitated DNA (recommended for Illumina library preparation), we generated up to 75 ng/μl (total volume 40 μl) final DNA library for sequencing (Table 4), which suggests that our method worked effectively for a range of histone proteins. Chromatin DNA recovery is largely dependent on the ability of antibodies to immunoprecipitate its target protein [6], which could explain the difference in DNA concentration between antibodies.

**Table 3:** An example of fruit fly output DNA recovered after conjugation of specific antibodies when using known concentrations of cross-linked DNA.

Antibodies	*Bactrocera tryoni*		*Ceratitis capitata*		*Bactrocera dorsalis*		*Zeogodacus cucurbitae*	
	Cross-linked (ng)	Conjuga-ted (ng)	Cross-linked (ng)	Conjuga-ted (ng)	Cross-linked (ng)	Conjuga-ted (ng)	Cross-linked (ng)	Conjuga-ted (ng)
H3K36me3	248	80.55	212	73.48	-	-	-	-
H3K36me1	244	135.96	-	-	184	78.66	195	88.42
H3K4me3	302	54.74	-	-	-	-	-	-
H3K4me2	272	172.92	-	-	-	-	-	-
H3K27me3	340	93.72	-	-	162	64.43	163	72.95
H3K27ac	332	150.04	148	50.48	-	-	-	-
Pan-H3	250	66.24	133	51.98	174	52.64	192	71.28
Mock	312	< 0.05	147	< 0.05	168	1.01	183	< 0.05

**Table 4:** Output DNA of 200–300 bp size (enriched product) obtained from a starting material of 10 ng of antibody conjugated DNA from *Bactrocera tryoni* head tissues using Illumina Truseq ChIP sample preparation kit.

Histone antibodies	DNA concentration (ng/μl)	Total concentration (μg)
H3K36me3	60.2	2.41
H3K36me1	64	2.56
H3K4me3	59.4	2.38
H3K4me2	68.6	2.74
H3K27me3	60.8	2.43
H3K27ac	66.4	2.66
Pan-H3	75	3.00

**Fig 3:**
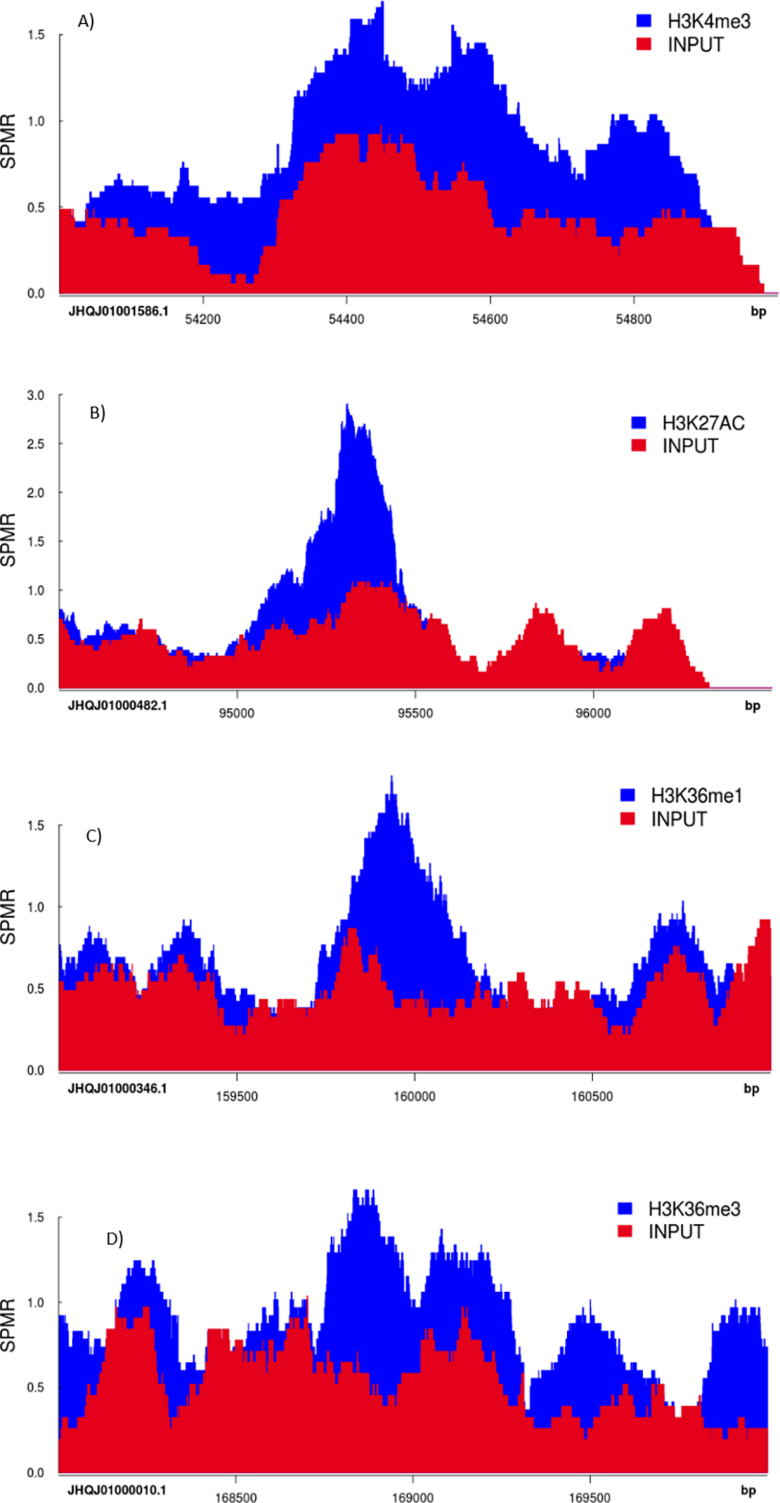
An example of genomic regions displaying enrichment for H3K4me3, H3K27Ac, H3K36me1 and H3K36me3 histone proteins in *Bactrocera tryoni* when visualised using the ‘Sushi.R’ R/Bioconductor package (SPMR—Sequences Per Million Reads). ‘Input’ samples that were not conjugated with any antibodies served as ‘control’ to compare histone modifications in antibody conjugated samples. Differential peak height and width represents enrichment of histone proteins at various genomic regions.

We noticed ‘daisy chain’ (S1 Fig. Daisy-chain—a larger, almost double the size, trailing peak noticed in bioanalyzer profile after enrichment) after enrichment of chromatin DNA when we employed 18 cycles of enrichment that was recommended in the Illumina TruSeq ChIP sample preparation method (Preheat the lid to 100°C; 98°C for 30 sec; 18 cycles of 98°C for 10 sec, 60°C for 30 sec, 72°C for 30 sec; 72°C for 5 min; hold at 4°C). This was perhaps caused by over-amplification; hence, starting the enrichment process with less cycles and gradually increasing the cycle can help optimize the PCR enrichment conditions. It should be noted, however, that the enrichment of DNA also depends on the efficiency of the antibody used to immunoprecipitate the target antigen. For sequencing, we used the Illumina NextSeq 500 platform, which produces approximately 400 million paired end sequencing reads per sequencing run (see Table 5 for example of sequencing output produced from the NextSeq 500 platform). We selected a read length of 40 bp per read (i.e. 80 base pairs per read pair), which was sufficient to align reads to the *B*. *tryoni* reference genome. After performing read alignment and, peak calling was carried out using MACS2 software, and genomic regions displaying enrichment for specific histone proteins were visualised using the ‘Sushi.R’ R/Bioconductor package [36]. Example peak data for histone proteins H3K4me3, H3K27Ac, H3K36me1 and H3K36me3 are presented in Fig 3, which evidences histone modification in various genomic regions of tephritids for the first time.

**Table 5:** Summary sequence data from a single Illumina run (several runs were performed for different antibodies).

Indexing QC							
	Total Reads	PF Reads	% Reads Identified (PF)		CV	Min	Max
Lane 1	233013360	219931326	97.9531		0.1607	4.951	8.4771
Lane 2	229440604	217221220	97.6945		0.1611	4.9513	8.4623
Lane 3	235203672	222668790	97.8653		0.1606	4.9515	8.4662
Lane 4	230079740	218419190	97.6604		0.1608	4.95	8.4513
Run metrics from all 4 lanes							
	Cycles	Yield	Projected Yield	Aligned (%)	Error Rate (%)	Intensity Cycle 1	%≥Q30
Read 1	40	17.13 Gbp	17.13 Gbp	0.98	0.18	8,611	96.91
Read 2	6	2.20 Gbp	2.20 Gbp	0.00	0.00	6,137	96.88
Read 3	40	17.10 Gbp	17.10 Gbp	0.96	0.22	9,103	94.61

The ChIP methodology described here allows performance of high throughput epigenetic studies and will advance the functional ‘omics’ research in tephritid fruit flies. It adds to the existing genomic and transcriptomic resources already available for tephritid fruit flies [28–34; 37–39], and will assist researchers to understand complex biological and behavioural processes in these economically important flies. Further, this method summarises key steps in ChIP that need optimization, and hence can aid others developing ChIP in non-model insects.

## Supporting information

S1 Fig Daisy-chain—a larger, almost double the size, trailing peak noticed in bioanalyzer profile after enrichment. (DOCX)

S1 Table List of reagents and equipment used for chromatin immunoprecipitation sequencing of tephritid fruit flies. (DOCX)
