# Supplementary figures and images for: Chromatin immunoprecipitation (ChIP) method for non-model fruit flies (Diptera: Tephritidae) and evidence of histone modifications
Source: PLoS One. 2018 Mar 15;13(3):e0194420. doi: 10.1371/journal.pone.0194420 (PMC5854383; doi:10.1371/journal.pone.0194420)

Figure S1.


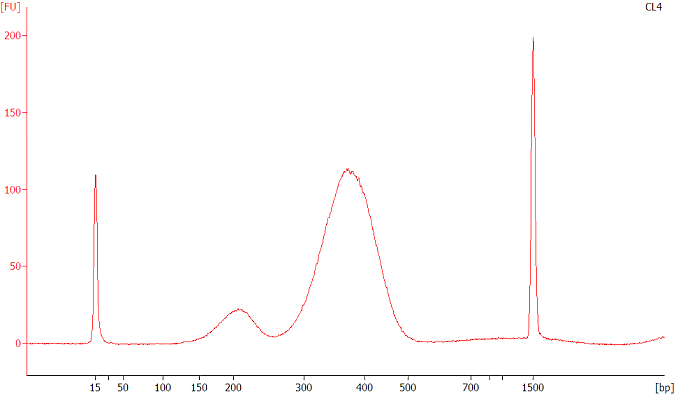


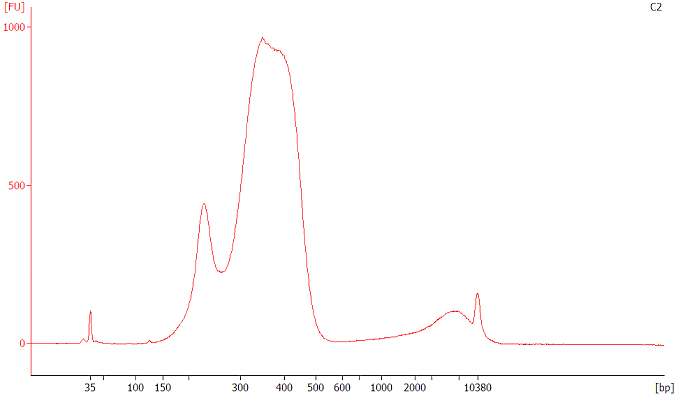

Supplement: S1 Fig — (DOCX) [file pone.0194420.s001.docx]
