# Supplementary material for: Chromatin immunoprecipitation (ChIP) method for non-model fruit flies (Diptera: Tephritidae) and evidence of histone modifications
Source: PLoS One. 2018 Mar 15;13(3):e0194420. doi: 10.1371/journal.pone.0194420 (PMC5854383; doi:10.1371/journal.pone.0194420)
Supplement: S1 Table — (DOCX) [file pone.0194420.s002.docx]

Table S1. List of reagents and equipment used for chromatin immunoprecipitation sequencing of tephritid fruit flies.

| *Cross-linking and immunoprecipitation* | *Library preparation* |
| --- | --- |
| 1. Phosphate buffered saline (DPBS) | 1. Illumina library preparation kit |
| 2. Protease inhibitor cocktail | 2. Illumina PCR kit |
| 3. Formaldehyde | 3. SYBR gold |
| 4. Glycine | 4. Ampure XP beads |
| 5. Ribonuclease A |  |
| 6. Proteinase K | *Equipment* |
| 7. Protein A/G beads (ChIP grade) | 1. Bioruptor® UCD-200 |
| 8. Hering Sperm DNA (HS DNA) | 2. Water bath |
| 9. Bovine serum albumin (BSA) | 3. Refrigerated centrifuge |
| 10. Antibodies of interest (ChIP grade, Table 2) | 4. Rotator |
| 11. Sodium Chloride | 5. Thermocycler |
| 12. Sodium deoxycholate | 6. Electrophoresis unit and reader |
| 13. Sodium bicarbonate | 7. UV illuminator for gel excision |
| 14. Calcium Chloride | 8. Illumina Nextseq 500 |
| 15. Sodium Butyrate | 9. Realtime PCR |
| 16. Tris Hydrogen chloride | 10. Bioanalyser |
| 17. Triton X-100 | 11. Qubit |
| 18. Ethylene diamine tetra acetic acid (EDTA) |  |
| 19. Sodium dodecyl sulphate (SDS) |  |
